# Effect of Artificial Piglet Suckling Sounds on Behavior and Performance of Piglets and Adrenal Responses of Sows

**DOI:** 10.1155/2018/2762153

**Published:** 2018-10-22

**Authors:** Jaruwan Khonmee, Thanat Wathirunwong, Terdsak Yano, Chaleamchat Somgird, Janine L. Brown, Panuwat Yamsakul

**Affiliations:** ^1^Department of Veterinary Bioscience and Veterinary Public Health, Faculty of Veterinary Medicine, Chiang Mai University, Chiang Mai 50100, Thailand; ^2^Excellent Center in Veterinary Bioscience, Chiang Mai University, Chiang Mai 50200, Thailand; ^3^Department of Food Animal Clinic, Faculty of Veterinary Medicine, Chiang Mai University, Chiang Mai 50100, Thailand; ^4^Department of Companion Animal and Wildlife Clinic, Faculty of Veterinary Medicine, Chiang Mai University, Chiang Mai 50100, Thailand; ^5^Center for Species Survival, Smithsonian Conservation Biology Institute, Front Royal, VA 22630, USA

## Abstract

Operation of the farrowing house is essential to the productivity of a swine farm, requiring not only good management but also knowledge of the behavior of sows and piglets. Stress can negatively affect production in farm animals and could be a factor in production indexes. The objective of this study was to investigate the effect of artificial sucking sounds on the behavior of piglets and fecal glucocorticoid (FGM) concentrations of sows. A total of 30 sows were divided into two groups: a treatment group (15 sows) was exposed to artificial sucking sounds and a control group (15 sows) was not. Both groups received the same management; the two open-house system locations were separated by a distance of about 270 meters. The study had three key objectives: to compare farrowing indexes and to observe the sucking behavior of piglets using CCTV cameras. Fecal samples were collected daily for 21 days from the period after parturition to weaning to assess adrenal activity. The treatment group had a significantly higher average number of times piglets came to a sow's udder, and sows had a shorter onset time for the first piglet to come to the sow's udder than the control group (both* P<0.05*). The patterns and levels of FGM between the two groups were not different (both* P<0.05*), but the treatment group had better farrowing indexes than the control group (*P>0.05*), particularly for litter weight gain and percent preweaning mortality. In addition, the weaning to first service interval of the treatment group was shorter than the control group (*P<0.05*). This indicates that the artificial suckling sound probably has no adverse effect on adrenal responses of pig; however, it improves production indexes of postparturition sows.

## 1. Introduction

Good production indexes on a swine farm are dependent on good management in the farrowing house. One factor in that index is the quantity and quality of milk provided to piglets by nursing sows which affects the strength of the piglets and their potential as they mature. Related to that is the milk let-down reflex of sows which depends on many different hormones, especially prolactin and oxytocin [1–3]. Both of those hormones are stimulated by piglets massaging the udder [4]. Each cycle of milk let down lasts approximately 40 to 60 minutes and begins about 1 to 3 minutes after massage by a piglet begins [5, 6].

Normally, sounds made by the sow can induce the suckling behavior of piglets including massaging the sow's udder; i.e., sound is important for stimulating suckling behavior of piglets. Kasanen and Alger [7] reported that pulsating sounds made by sows can induce suckling behavior in piglets [4]. If piglets can receive sow's milk in a higher level, they will be resulting in a better production performances e.g., increased weaning weight, increased number of piglets weaned per sow, and decreased preweaning mortality.

Previous studies have demonstrated a relationship between stress and suckling sounds [2, 8, 9]. Major stressor would be negatively affecting sow performances if suckling sounds are played all day round [8]. The higher level of 90 decibels of suckling sounds would lead a deviant of sow feeding patterns and other maternal behaviors [2] which are negatively impact on the condition of sows after lactating period e.g., an increased weaning to first estrous interval (WFEI) [9]. All of which can increase glucocorticoid production from the adrenal glands [2, 8, 9]. Chronic glucocorticoid exposure can then lead to problems, including abnormal behavior, suppressed immune function, poor population performance, and disruption of reproductive hormone production [9–11]. One widely used method to monitor stress responses is through the analysis of adrenal steroid hormone metabolites excreted in urine and feces [12]. Rather than traditional methods of blood collection, noninvasive glucocorticoid monitoring is now well established as a valuable tool for evaluating adrenal gland activity in various ungulate species [13–15].

In this study, we hypothesized that artificial suckling may affect both behavior and also stress hormone. Specifically, the goal was to determine the effects of artificial suckling (AS) sound on the suckling behavior of piglets and sows. Thus, the aim of this study was to (1) observe suckling behavior of piglets; (2) evaluate the production indexes of piglets and sows during the lactating period; (3) to assess adrenal responses of sows.

## 2. Materials and Methods

### 2.1. Animals

This study was approved by the Faculty of Veterinary Medicine, Chiang Mai University, Animal Care and Use Committee (FVM-ACUC) (Permit Number S017/2559). A total of 30 Large White X Landrace sows (aged 12-36 months, weighing 150-300 kg) with a combined 300 piglets were housed at the KS Farm, Khon Kaen in Thailand. Sows were maintained in individual farrowing crates and fed* ad libitum* twice daily with a pelleted ration (1.5 kg, Betagro 004 farrowing pellet [18% protein, 3% fat, 12% fiber, and 13% moisture], Betagro Company Limited, Thailand) with unlimited access to fresh water. Animals were given annual physical examinations by the staff veterinarian and were dewormed every 3 months. All sows were in good health during the study.

### 2.2. Experimental Design

Animals were divided into two groups: a treatment group (n=15) that was exposed to AS sounds and a control group (n=15) that was not. The sample size was calculated using the G-power program with effect size, alpha error probability, and power of 0.05, 0.05, and 0.95, respectively. Pigs in the treatment group were exposed to AS sounds (approximately 70 decibels) for about 8 minutes every 35 minutes over a 24-hour period for 21 days from the period after parturition to weaning. The behavior of the piglets and sows in the farrowing pens was monitored by CCTV cameras 24 hours a day until weaning (21 days). Both groups had the same management, feed, and housing situation and were separated from each other by about 270 meters. There was no significant difference between the groups in the fundamental production indexes (*P>0.05*) (Table 1).

### 2.3. Production Indexes

Production indexes of sows and piglets included the weaning to first estrous interval, birth weight, litter size, number of live births, weaning weight, average number of pigs weaned per sow, litter weight gain, number of piglets lost, and also percentage of preweaning mortality that was recorded (Table 2).

### 2.4. Behavioral Observations

Fifteen sows in each group were monitored 24 hours per day for 21 days starting on the day of farrowing by CCTV cameras to assess the behavior of the piglets, including the number of times that >80% of the piglets in the litter came to the sow's udder and the onset time (minutes from the onset of the AS sound until the first piglet came to the sow's udder).

### 2.5. Fecal Sample Collection and Processing

Fresh fecal samples (~30 g) were collected into ziplock plastic bags between 08:00 and 09:00 hours from 10 of the sows from farrowing to weaning by rectal collection, daily for 21 days while doing the experiment Samples were frozen immediately and stored at -20°C until being extracted for FGM. All chemicals were obtained from the Sigma Chemical Company (St. Louis, MO) unless otherwise stated. Following the protocol of Khonmee et al. [16], wet fecal samples were dried in a conventional oven at 60°C for 24 hours and stored at -20°C until extraction. Frozen dried fecal samples were thawed at room temperature, mixed well, and pulverized, and (0.2 ± 0.01) g was boiled in 5 ml of 90% ethanol:distilled water for 20 min [16] After centrifugation at 3500 × g (20 min), the supernatant was recovered and the pellet resuspended in 5 ml of 90% ethanol:distilled water, vortexed for 1 min, and recentrifuged (3500 × g, 20 min) [16]. The extraction was performed twice and the two supernatants were combined, dried down under air in a warm water bath (50°C), and reconstituted in 1 ml dilution buffer (0.1 M NaPO4, 0.149 M NaCl, pH 7.0) [16]. The extracts were stored at -20°C until further analysis. The efficiency of extraction of steroid from feces was 91.8% based on the recovery of corticosterone standard added to the dried fecal samples prior to extraction. The corticosterone EIA was validated for pig fecal extracts by showing a significant increase in concentrations in sows after weaning, comparing daily fecal samples from 7 days before and after weaning.

### 2.6. Enzyme Immunoassays

FGM metabolites were quantified using a double-antibody enzyme immunoassay (EIA) [17] that relied on a polyclonal rabbit anti-corticosterone antibody (CJM006) as described by Khonmee et al. [16]. Plates were coated with anti-rabbit IgG (10 *μ*g/ml; Cat. No. A009, Arbor Assays, Ann Arbor, MI) in coating buffer (Cat. No. X108, 20X, Arbor Assays, Ann Arbor, MI) by adding 150 *μ*l to each well of a 96-well microtiter plate (Cat No 07-200-39, Fisher Scientific, Pittsburgh, PA) followed by incubation at room temperature (RT) for 15-24 hours for the corticosterone EIA [16] The contents of the wells were emptied, the plates were blotted dry, and blocking solution (Cat. No. X109, 10X, Arbor Assays, Ann Arbor, MI) was added to each well (250 *μ*l) and incubated for 15-24 hours at RT Following incubation, the contents of the wells were emptied, and the plates were blotted and dried at RT in a Dry Keeper (Sanplatecorp, Osaka, Japan) with loose desiccant in the bottom [16] After drying (humidity <20%), plates were heat sealed in a foil bag with a 1 g desiccant packet and stored at 4°C until use.

Using the procedure of Khonmee et al. [16], antibody-coated plates were brought to RT, and EIA buffer (0.1 M NaPO4, 0.149 M NaCl, 0.1% bovine serum albumin, pH 7.0) was added to the nonspecific binding (75 *μ*l; NSB) and maximum binding (50 *μ*l) wells Corticosterone (50 *μ*l, range 3.9 -1,000 pg/well; C2505 Sigma-Aldrich, Dorset, UK) standards and samples diluted in EIA buffer (50 *μ*l, 1:30 dilution for FGM) were combined with steroid-HRP (25 *μ*l; 1:30,000 dilution for corticosterone, U.C. Davis, CA) followed by addition of 25 *μ*l of primary antibody (1:100,000 dilution for corticosterone), except NSB wells, and incubated at RT for 1 hour [16]. Plates were washed four times with wash buffer before addition of 100 *μ*l of TMB substrate solution (Ward Medic, Bangkok, Thailand). After incubation for 45-60 min at RT without shaking, the absorbance was measured at 620 *μ*m (TECAN Sunrise™ microplate reader, Salzburg, Austria) until the optical density approached 0.9 and then stop solution (0.16 M sulfuric acid) was added (50 *μ*l) to each well [16]. The absorbance was measured at 405 *μ*m (TECAN Sunrise™ microplate reader, Salzburg, Austria.

(The EIAs were validated for sow feces by demonstrating parallelism between serial dilutions of pooled extracts and the corticosterone standard curve (Pearson's correlation coefficient for FGM, r = 0.99). Addition of unlabeled standard to pooled fecal extracts before extraction resulted in significant mass recoveries (y = 0.27x – 0.35, R^2^ = 0.94,* P* < 0.05). Extraction efficiencies were 80-90%. Assays were biologically validated by showing a marked increase in FGM concentrations in a sow that was restrained by the animal care taker. Assay sensitivity was 0.078 ng/ml at 90% binding Interassay CV was <15% based on binding of high (30%) and low (70%) control samples All samples were reanalyzed if the duplicate CVs were >10%; thus, intra-assay CV was <10%. Data are expressed as ng/g dry feces.

### 2.7. Statistical Analysis

The parameters of farrowing indexes and averages of piglet behavior of both groups were compared and analyzed by t-test using SPSS v21.0 (*P<0.05*). Those parameters were composed of litter size, number born alive, pigs weaned per sow, average weaning weight, litter weight gain, and weaning to first estrous interval.

FGM concentrations were averaged for each of the three time periods, 0-7, 8-14, and 15-21 days after parturition. The data were analyzed using a general linear model (GLM) and repeated ANOVA. P values<0.05 were considered statistically significant.

## 3. Results

Most production indexes did not differ significantly between treatment and control groups (*P>0.05*), with the exception of weaning to first service interval, which was shorter in the treatment compared to the control group (*P=0.02*) (Table 2).

For comparison of the number of times that 80% of the piglets came to the sow's udder to suckle during the lactation period, in the treatment group (exposed to AS sounds) it was was greater and averaged 10.26±0.32 times compared to the control group (not exposed to AS sounds) that only averaged 5.07±0.29 times (*P<0.05*). Moreover, onset time of the first piglet coming to the sow's udder to suckle was shorter in the treatment group (3.88±0.05 minutes) compared to the control group (4.35±0.07 minutes) (*P<0.05*).

**Figure 1:**
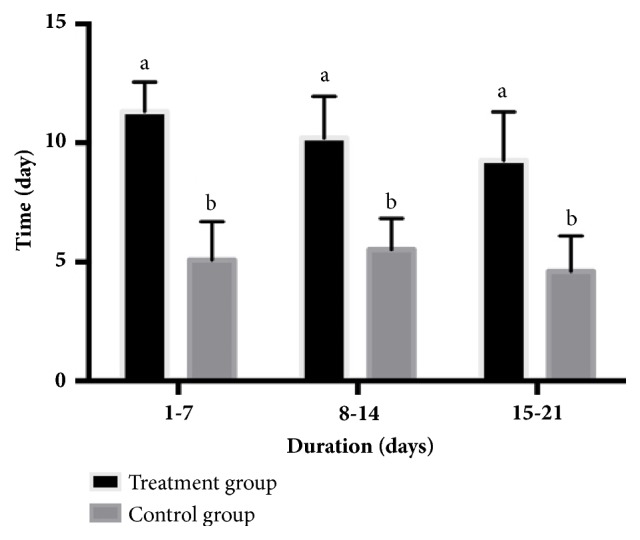
Comparing the number of times 80% of the piglets came to sow's udder to suckle in the treatment group (exposed to AS sounds) and the control group (not exposed to AS sounds) in each lactating period (1-7, 8-14, and 15-21 days after parturition). ^a,b^Statistical significance (P<0.05) between groups.

**Figure 2:**
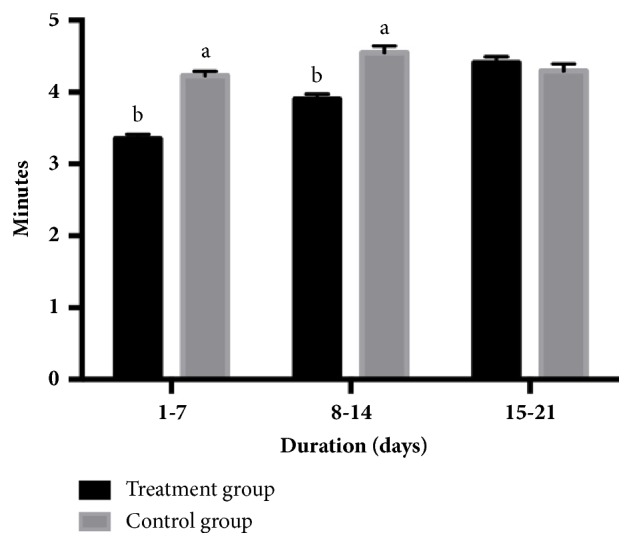
Comparison of onset time for the first piglet to come to the sow's udder to suckle in the treatment group (exposed to AS sounds) and the control group (not exposed to AS sounds) in each lactating period (1-7, 8-14, and 15-21 days after parturition). ^a,b^Statistical significance (P<0.05) between groups.

The number of times 80% of the piglets came to sow's udder to suckle is illustrated in Figure 1, and the onset time for the first piglet to come to the sow's udder to suckle is illustrated in Figure 2. Data are shown as three separate 7-day intervals throughout lactation (1-7, 8-14, and 15-21 days). The number of times 80% of the piglets came to sow's udder to suckle for the treatment group was higher than the control group in all periods (*P<0.05*). By contrast, the onset time for the first piglet to come to the sow's udder to suckle was only higher during the first two lactation periods (1-7 and 8-14 days) in the treatment group (*P<0.05*).

**Figure 3:**
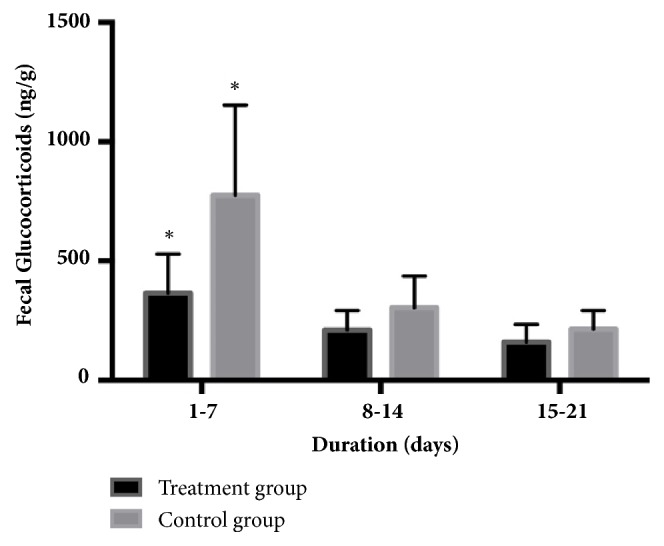
Cortisol level in fecal matter of sows in the treatment group (exposed to AS sounds) and the control group (not exposed to AS sounds) in each lactating period (1-7, 8-14, and 15-21 days after parturition). *∗*Statistical significance (*P<0.05*) within groups.

Figure 3 showed that the concentrations of FGM were higher in control versus treatment groups during the early lactation period (days 1-7) only, when compared to the other time periods (days 8-14 and days 15-21) (*P<0.05*). However, FGM were not influenced significantly by AS during the other lactational periods and overall were lower than during early lactation.

**Figure 4:**
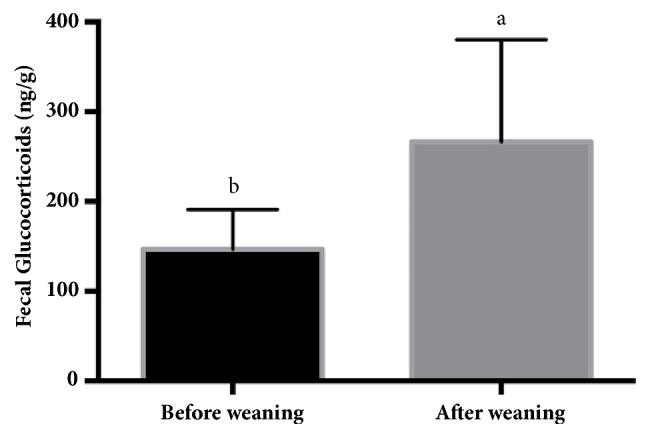
Comparison of fecal glucocorticoid metabolite concentrations (mean±SD) between preweaning and postweaning sows. ^a,b^Significant difference (*P<0.05*). *∗*Statistical significance (*P<0.05*) between groups.

Biovalidation was used to compare FGM concentrations in the preweaning and postweaning periods (Figure 4). The preweaning period was considered to be a nonstress condition and postweaning period was considered to be a stress condition. The results show that FGM concentrations of preweaning sows were lower than in postweaning sows (*P<0.05*), confirming the validity of the stress evaluation.

### 3.1. Discussion and Conclusions

The results indicate that the AS sounds stimulated the suckling behavior of piglets and resulted in improvement in some production indexes including litter weight gain, average pig weaning weight per sow, and percent preweaning mortality. In addition, the weaning to first service interval of the treatment group had significantly differenced from what was significantly shorter than the control group (*P<0.05*), indicating that sows exposed to AS sounds have greater fertility efficiency than sows not exposed to AS sounds. Importantly, the high frequency artificial sucking sound at 70 decibels does not cause stress in sows.

**Figure 5:**
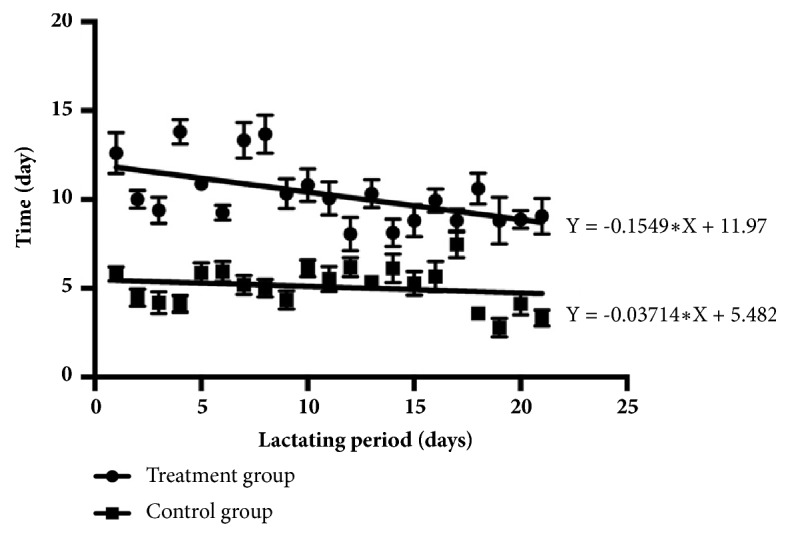
Frequency of times piglets came to a sow's udder in the treatment group (exposed to AS sound) and the control group (not exposed to AS sound) each day of the lactation period.

**Figure 6:**
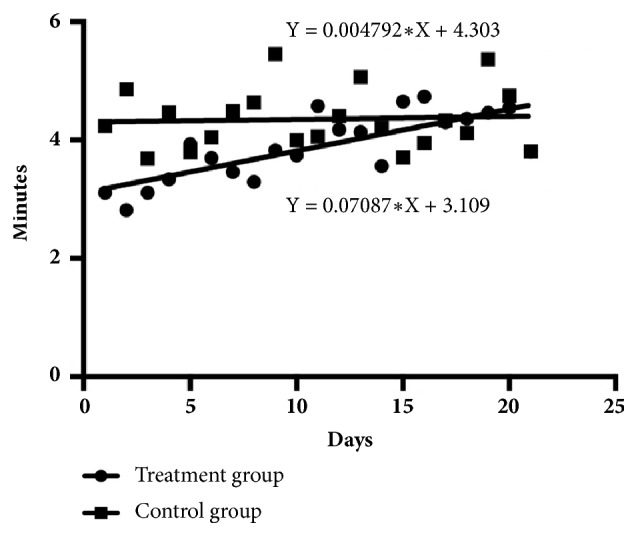
Onset time of the first piglets coming to the sow's udder to suckle after exposure to the suckling sounds (treatment group) and the control group (not exposed to the AS sound) each day of the lactation period.

Previous studies have reported that the grunting sounds of sows can stimulate the suckling behavior of piglets [7] and that AS sounds made by humans can also stimulate that behavior in piglets [18]. When piglets were exposed to AS sounds for the first time after parturition, the sounds were recognized and responded to normally [19]. The suckling behavior was stimulated by sound the piglets responded to; however, that behavior decreased as the weaning period neared because of many factors such as consumption of other foods (creep feed) [20]. Linear regression was used to analyze the frequency at which piglets came to a sow's udder when exposed to AS sound (treatment group) and when not exposed to AS sound (control group) each day of the lactating period. The regression line for the treatment group was Y=-0.1549X+11.97 and for the control group was Y=-0.03714X+5.482. The treatment group regression line had a steeper slope compared to the control group (Figure 5). In addition, the onset time of the first piglet coming to the sow's udder after hearing the suckling sound in the treatment group and in the control group where no suckling sound was heard was recorded for each day of the lactating period. The regression line for treatment group was Y=0.07087X+11.97 and for the control group was Y=0.004792X+4.303. The treatment group had a steeper regression line slope than the control group (Figure 6). All results supported the reason above that the piglet of treatment group had a higher decrease to response behavior than the piglet of control group with the continuing time to nearby weaning period. Response behavior of treatment group piglets declined more rapidly than that of control group piglets toward the end of the weaning period.

It has been demonstrated that glucocorticoid metabolites appear in fecal matter approximately 48 hours after exposure to stressful conditions [21]. The level of glucocorticoid metabolite hormone has been shown to increase with the level of severity of the stress [22]. The AS sound did not simulate stress in the sows (Figure 3) as evidenced by the fact that both groups had the same patterns of FGM concentrations. In our study, the FGM concentration during days 1-7 after parturition was higher than during the next two periods (8-14 and 15-21 days after parturition) (Figure 3). That indicates that both groups were exposed to equal levels of stress from parturition through each 7-day period following parturition. Overall, the FGM concentration 1-7 days after parturition in control group was higher than the treatment group. Control group may have been exposed to other handling or environmental stressors [23], but since both groups were treated the same, it could be that the AS sounds were soothing to the treated pigs. Thus, it can be concluded that neither the AS sound at 70 decibels nor the higher frequency of piglet suckling in the treatment group resulted in additional stress for the sows.

One surprising finding in this study was that the weaning to first service interval of the treatment group was significantly shorter than the control group* (P<0.05)*. Ideally, sow fertilities are negatively associated with wean to first estrous intervals [24]. However, higher frequencies and long periods of piglet sucklings time might be a factor corelating to the longer wean to first estrous interval [25]. In this study, the opposite relationship was observed: the weaning to first estrous interval of the treatment group was shorter than that of the control group. One possibility is the increased sucking stimulus in the treated pigs caused an increase in *β*-endorphin hormone release [26], which increases when piglets suck the sow's udder [27]. This hormone helps to decrease pain and inhibit the release of stress hormones [28]. Weaning to first estrous period also depends on the feed intake of the sow during the lactating period and the quantity of milk consumed by the piglets, but these two groups were fed the same.

In conclusion, the AS sound can stimulate the suckling behavior of piglets, especially in days 7-14 after parturition, but the sound had no adverse effect on the stress level of the sows. Additional studies are needed to examine feed intake of the sows, the duration of the suckling period for each piglet during each suckling event, and increasing time for experiment in sow until results after service. Moreover, further studies of nursery pigs also are warranted.

## Figures and Tables

**Table 1:** Comparison of fundamental production indexes between control group (no suckling sound; n=15) and treatment group (suckling sound; n=15) (mean±SD).

Indexes	Control group	Treatment group	P-value
Parity	5.86±2.78	5.08±3.43	0.21
Litter size (piglets/sow)	13.11±2.77	12.37±3.31	0.22
Live births (piglets/sow)	12.81±2.82	12.04±3.14	0.19
Average birth weight (kg.)	1.45±0.25	1.43±0.19	0.69
Average lactating period (days)	21.00±0.00	20.91±0.45	0.21

**Table 2:** Production indexes of the control group (no suckling sound; n=15) and treatment group (suckling sound; n=15) (mean±SD) also.

Indexes	Control group	Treatment group	P-value
Total weaning weight per sow (kg)	55.86±13.57	59.05±10.99	0.20
Average weaning weight per sow (kg)	5.23±0.94	5.49±0.81	0.13
Pigs weaned per sow (number of pigs)	10.66±1.79	10.77±1.47	0.73
Litter weight gain (kg/day)	1.92±0.53	2.07±0.42	0.11
Pre-weaning mortality (number of pigs)	2.97±2.34	2.38±2.28	0.26
Pre-weaning mortality (%)	20.77±14.08	16.19±13.65	0.14
Weaning to first service interval (days)	5.35±2.76	4.41±0.82	0.02

## Data Availability

The data used to support the findings of this study are available from the corresponding author upon request.
